# A review on biological and chemical diversity in Berberis (Berberidaceae)

**DOI:** 10.17179/excli2014-399

**Published:** 2015-02-20

**Authors:** Sharad Srivastava, Manjoosha Srivastava, Ankita Misra, Garima Pandey, AKS Rawat

**Affiliations:** 1 Pharmacognosy and Ethno pharmacology Division, CSIR-National Botanical Research Institute, Lucknow-226001, India

**Keywords:** Berberis, berberine, pharmacology, ethno botany

## Abstract

*Berberis *is an important genus and well known in the Indian as well as European systems of traditional medicine. It is used since ancient times for curing eye disease, fever, jaundice, rheumatism, vomiting during pregnancy, kidney and gall balder stones and various other ailments due to the presence of biologically active alkaloid berberine. Action of the root extracts of few species are believed to be as powerful as quinine in the treatment of malarial fever.

A plethora of literature pertaining to the taxonomy, biology, chemistry, traditional and ethnic uses of *Berberis* in different countries and indigenous cultures was collected by both offline (library, journals, textbooks etc.) and online mode (electronic search of available databases). In addition to this, books on traditional medicine and ethno pharmacological knowledge were also referred to extract ancient uses of *Berberis* in different traditional medicine systems. Most of the folklore, traditional and ethno botanical claims about *Berberis* species were validated by broad spectrum *in vitro* and *vivo* pharmacological studies. The present article summarizes its usage in eye and liver disorder, fever, kidney and gall stones along with anticancer activity.

This comprehensive review will not only help researchers for further evaluation but also provide substantial information for future exploitation of species to develop novel herbal formulations.

## Introduction

The genus *Berberis *has an important place in various traditional systems of medicine worldwide for their efficacious medicinal properties. The ancient Ayurvedic literature of India records uses of *Rasaut* (*Ras *= juice; *out *= frothing and foaming when boiling; hence *Rasaut *means concentrated juice), an extract of either stem or root of *Berberis* sp. The specific uses of *Rasaut *for curing eye diseases and indolent ulcers earned a great fame. In the British Pharmacopoeia, the alkaloid berberine- the active principle in several species of *Berberis* and *Mahonia*, has been incorporated for its successful use in the treatment of oriental sore.

The roots of *Berberis* species are employed as an anti-periodic, diaphoretic and antipyretic, and its action was believed to be as powerful as quinine. The bark is used as a tonic and anti-periodic. This plant is also well proven for cardio vascular, hepato-protective, antimicrobial and anti-cancerous activities. Hence, a review of genus *Berberis* has been done to put all its activities, ethno botanical claims, pharmacological action along with chemistry. The scientific information compiled in this review is gathered by extensive search of several electronic databases *viz*. SCOPUS, Google scholar, NOPR, Pub med, Elsevier, ACs, Medline plus, Web of science, etc. Additionally, the library search and ancient medicinal treatises/text books were also referred for gathering information on the traditional uses of *Berberis*. The review of *Berberis* was also done with an end in view of identifying the knowledge gaps in traditional uses, pharmacological studies, toxicity proﬁling, clinical trials and other relevant research in this medicinally important genus. Previous reviews on individual species of *Berberis* are available, but a comprehensive update on the entire genus is still lacking. This review will help researchers to identify the latent and patent potentials of *Berberis* and explore further studies on the biological and chemical properties of various species of this genus.

## Taxonomic history of Berberis

*Berberis *belongs to the family Berberidaceae, which was first established by A.L. Jussieu in 1789 as 'Berberides' and was considered one of the most primitive families of Angiosperms having a high number of disjunction or discontinuous genera. There is a general agreement among botanists (Kumazawa, 1938[71]; Hutchinson, 1959[56]; Airy Shaw, 1966[4]; Takhtajan, 1969[127]; Meacham, 1980[84]; Nowicke and Skvarla; 1981[93]) that the genera of Berberidaceae are not closely related but are separable into 4 distinct families, namely Lardizabalaceae (*Decaisnea*, *Holboellia, Parvatia*), Nandinaceae (*Nandina*), Podophyllaceae (*Podophyllum*) and Berberidaceae (*Berberis*, *Mahonia*, *Epimedium*). Berberidaceae was placed in the order Ranales (Bentham and Hooker, 1862[11]). Several other works (Takhtajan, 1969[127]; Cronquist, 1968[28]) placed it in the order Ranunculales, while one worker (Hutchinson, 1959[56]) included this family under a separate order Berberidales.

First taxonomic account of the family Berberidaceae for the Indian subcontinent (Hooker and Thomson, 1875[49]) included six genera and 17 species. A revision of the genus *Berberis *was made by Schneider during 1905 and 1908 and recorded 13 new species and one variety from Indian region. Subsequently (Schneider, 1942[107]) a monograph of section *Wallichianae *was published in which he recognized 71 species in 8 subsections. Chatterjee (1953[22]) included 68 species of *Berberis*, 11 species of *Mahonia*, one species of *Epimedium* and two species of *Podophyllum*. In a survey (Ahrendt, 1941/45[2]) the *Berberis* spp. from Bhutan, Assam, South Tibet, Upper Burma and Northwest Yunnan and later (Ahrendt, 1961[3]) published a detailed revision of *Berberis *and* Mahonia* species. He included 52 species with 43 infra specific categories under *Berberis* and 11 species under *Mahonia* from the Indian region. It was again revised by including one new species (*B. victoriana*) from the Indian region (Chamberlain and Hu, 1985[17]). Jafri (1975[59]) while dealing with the Berberidaceae for the *Flora of West Pakistan *included only one species of *Mahonia* and 15 species of *Berberis* from Kashmir region. In a more recent study (Rao and Hajra, 1993[104]) while revising the family for the *Flora of India* included 54 species of *Berberis, *one species of *Epimedium* and 13 species of *Mahonia* from the present political boundaries of India. 

**Figure 1:**
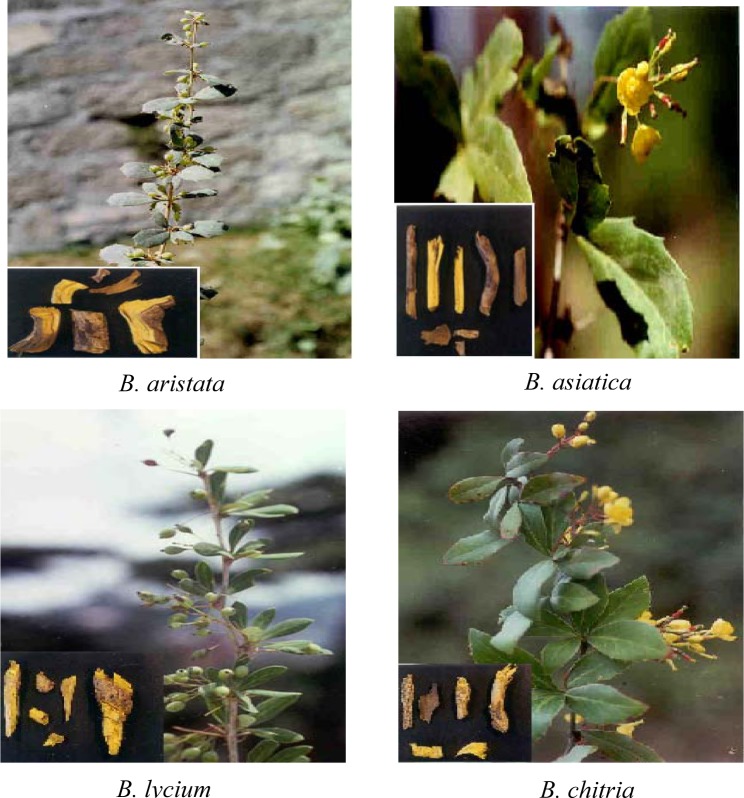
Some important species of genus *Berberis *collected from India

Singh et al. (1974[115], 1978[116]) discussed the significance of epidermal structure and leaf architecture in the taxonomy of Berberidaceae. They have studied hardly 5-6 species of the family. Palynologically only five species of *Berberis* have been studied (Nair, 1965[91]) and chromosome numbers in only nine species with five infraspecific categories of *Berberis* and three species of *Mahonia* have been reported (Kumar and Subramaniam, 1986[70]). Some commercially important *Berberis* spp. from Indian region is shown in Figure 1(Fig. 1).

## Ethnobotanical and traditional uses

There has been an increasing interest towards the scientific study of human-plant interaction in the natural environment among the botanists, social scientists, anthropologists, practitioners of indigenous systems of medicine. Jain (1981[60]) undertook intensive field study among tribes of central India and devised methodology for ethno- botany, particularly in the Indian context. Different species of genus Berberis are used ethno botanically and medicinally by various tribes and in different traditional medical systems. A detailed pharmacognostic study of some common Himalayan Berberis species has been done by Srivastava et al., 2001[125], 2004[122], 2006[124], 2010[123]; Singh et al., 2009[113], 2012[114]; Srivastava and Rawat, 2013[120], 2014[121]. The ethno botanical uses of Berberis by different tribal communities in India and some other countries are provided in Table 1(Tab. 1) (References in Table 1: ***B. aristata:*** Kirtikar and Basu, 1935[67]; Anonymous, 1948[9]; Hayashi, 1950[48]; Küpeli et al., 2002[72]; Uniyal, 1964[137]; Shah and Joshi, 1971[108]; Chauhan et al., 1978/79[25]; Jain and Suri, 1979/80[60]; Mittre, 1981[87]. ***B. asiatica:*** Bhattacharjee et al., 1980[14]. ***B. lycium:*** Koetz and Walter, 1979[68]; Virjee et al., 1984[139]. ***B. petiolaris:*** Bhargava, 1959[13]. ***B. tinctoria:*** Abraham, 1981[1]. ***B. wallichiana:*** Pal, 1984[94]. ***B. jaeshkeana***: Gaur et al., 1976[41]. ***B. vulgaris:*** Speck, 1998[119]; Tantaquidgeon, 1928[128]; Carr and Carlos, 1945[16]; Chandler et al., 1979[19]; Chaudhary et al., 1980[24]. ***B. holstii:*** Maliwichi-Nyirenda, 2011[82]).

## Pharmacological activities of Berberis species

**Figure 2:**
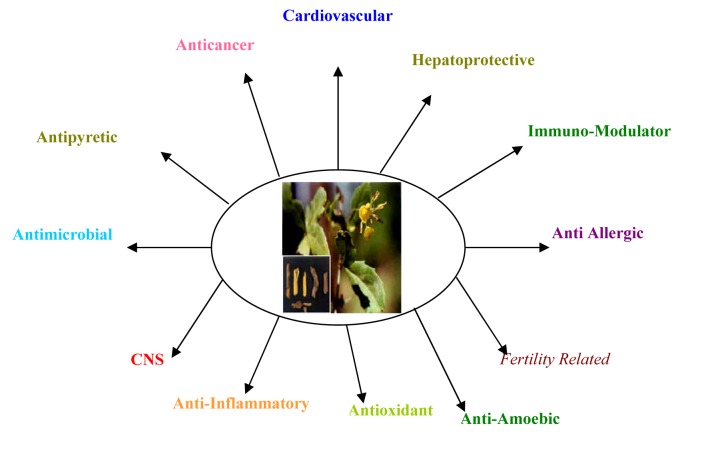
A broad spectrum of pharmacological activities of *Berberis*

Berberis has diverse pharmacological potential. Various pharmacological activities of the Berberis species make them an important part of polyherbal formulations for the treatment of several diseases and disorders (Figure 2(Fig. 2), Table 2(Tab. 2)) (References in Table 2: **Cardio vascular activity: *B. darwinii****:* Habtemariam, 2011[46]. ***B. aristata****: *Yogesh et al., 2011[148]. ***B. lycium****:* Fang et al., 1986[37]; Wang et al., 1987[142]; Neto, 1993[92]; Wang et al., 1993[141]. ***B. orthobotrys****:* Li et al., 1985[77]; Fang et al., 1986[37]; Li et al., 1991[76]; Li et al., 1986[79]. ***B. chitria****:* Xiong and Fang, 1989[144]. ***B. chilensis****:* Morales et al., 1989[88]; Morales et al., 1993[89]; Han et al., 1990[47]. ***B. paraspecta****:* Wang et al., 2004[140]. **Anti-inflammatory activity**: ***B. aristata****:* Akhter et al., 1977[5]. ***B. vulgaris****:* Invanovska and Philipov, 1996[58]. ***B. crataegina****:* Yeilada and Küpeli, 2002[147]; Jiang et al., 2011[63]; Chen et al., 2012[26]; Lee et al., 2013[73]. **Central Nervous System activity: *Berberis sp****.:* Shanbhag et al., 1970[112]. **Anti-convulsionactivity: *B. integerrima****:* Hosseinzadeh et al., 2013[50]. **Anti-histaminic and anti-cholinergicactivity: *B. vulgaris****:* Shamsa et al., 1999[111]. **Anti-microbialactivity: *B. vulgaris****:* Sack and Frochlich, 1982[106]. ***B. chitria****:* Dobhal et al., 1988[30]. ***B. heterophylla****:* Freile et al., 2003[38]. ***B. aetnensis****:* Musumeci et al., 2003[90]. **Hepato protective activity: *Berberis sp****.:* Chan, 1977[18]; ***B. integerrima****:* Jamshidzadeh and Niknahad, 2006[62]; Domitrovic et al., 2011[31]. ***B. aristata****:* Sohni and Bhatt, 1996[117]. ***B. aristata****: *Sohni et al., 1995[118]. **Anti-cancer activity: *Berberis sp****.:* Fukuda et al., 1999[40],[39]. ***B. amurensis****:* Xu et al., 2006[145]. ***B. koreana****:* Qadir et al., 2009[99]. **Hepato-carcinoma: *Berberis sp****.:* Li et al., 2013[80]; Kim et al., 2012[66]; Chueh and Lin, 2012[27]. **Antipyretic activity: *Berberis sp****.:* Sabir et al., 1978[105]. **Immuno-stimulant activity: *Berberis sp****.:* Li and Sui, 1986[78]. ***B. koreana****:* Qadir et al., 2008[100]. **Fertility related activity: *B. vulgaris****:* Aliev and Yuzbashinskaya, 1953[7]. ***B. chitria****:* Gupta and Dixit, 1989[45]. **Anti-oxidantactivity: *Berberis sp****.:* Ju and Han, 1990[64]. **Anti-diabetic activity: *B. vulgaris****: *Rajaei et al., 2011[103]; Meliani et al., 2011[85]. **Urolithiasis:* B. vulgaris****:* Jyothilakshmi et al., 2013[65]; Bashir and Gilani, 2011[10]. **Osteolytic and Hyper choletrolemic: *B. aristata****: *Zhou et al., 2012[149]; Rahigude et al., 2012[102]; Dong et al., 2011[32]; Huang et al., 2012[51]).

## Chemical diversity in Berberis

**Figure 3:**
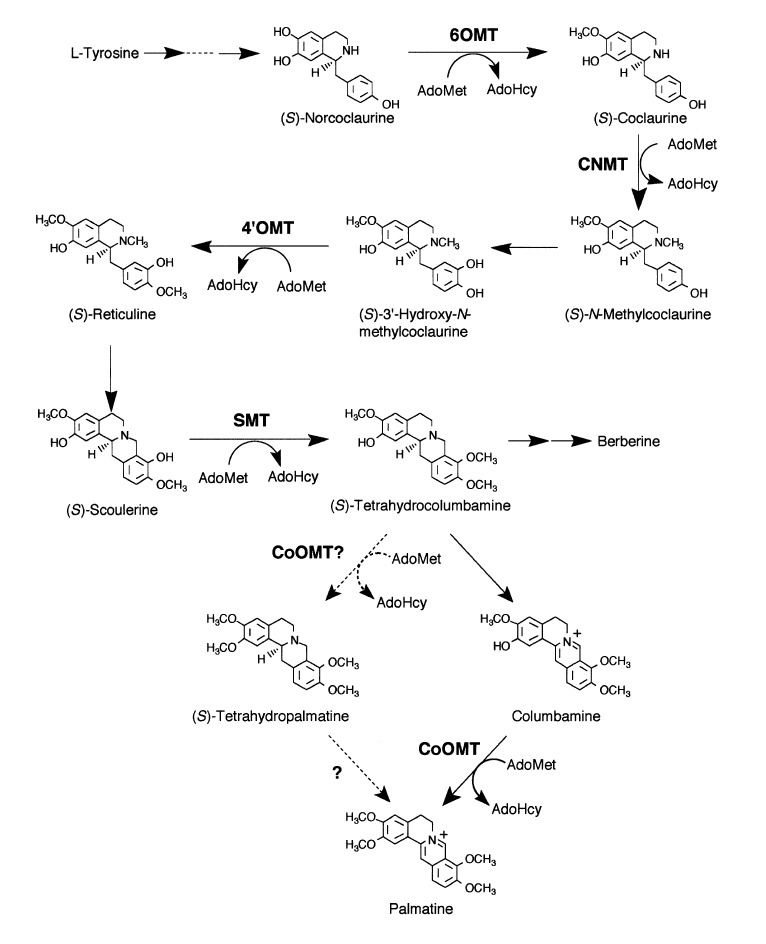
Biosynthetic pathway of Berberine and allied alkaloids

**Figure 4:**
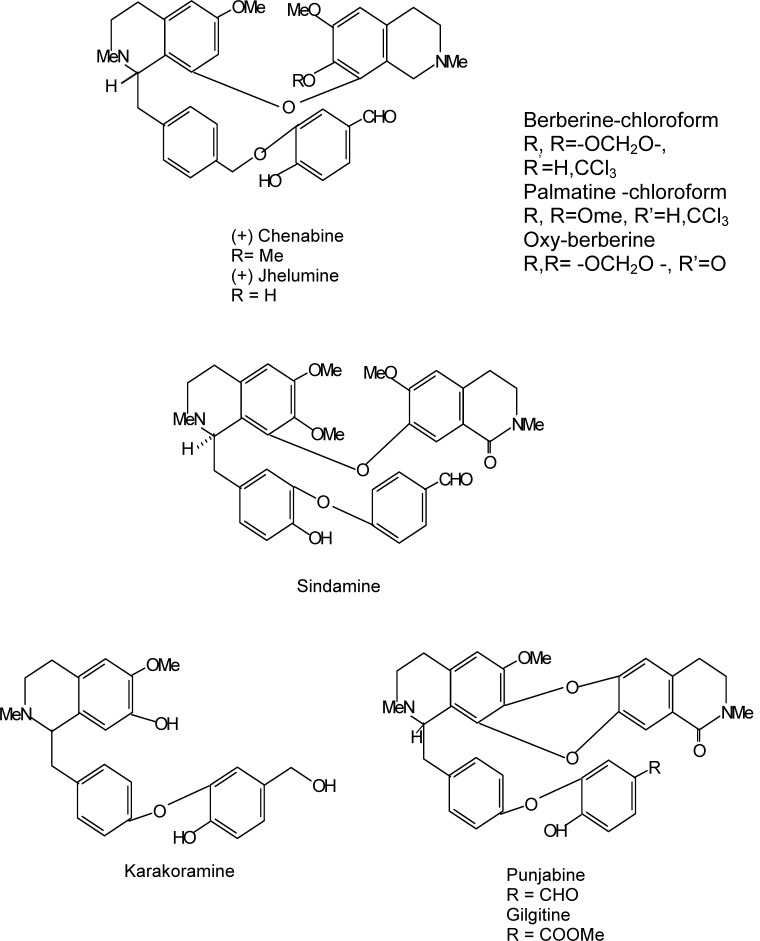
Some other important active principles of *Berberis*

Isoquinoline alkaloids are the major bioactive constituents in Berberis (Figure 3(Fig. 3)). Berberine is a major representative of the protoberberine alkaloids which are a structural class of organic cations, characteristically yellow, having four linked benzene rings with a nitrogen atom joining two rings pairs, and modified via two oxygen atoms at each end. A vast array of alkaloids has been isolated from various Berberis species, among which, berberine, berbamine, Palmitine, jatrorrhizine and isotetrandrine are the most common ones (Figure 4(Fig. 4)).

The histological distribution of berberine has been well studied; alkaloids of Berberis are located chiefly in the cortical tissues of the roots and stems. The bark of old roots contains the highest concentration of alkaloids. In the upper parts of the stem, concentration is low and in young leaves alkaloids could not be detected (Greathouse and Rigler, 1940[43]; Greathouse and Watkins, 1938[44]). Histological distribution of berberine, umbellatine and nepiotime has also been examined in Indian species of Berberis (Chatterjee, 1952[21]; Chatterjee et al., 1954[23]). Mean value of berberine content for young actively growing shoots is 0.04 % and for young parenchymatous roots is 1.41 %. Thus there is a progressive increase in the berberine content of the plants with an increase in age. 

Chemical analysis of the traditional preparation 'Rasaut' from Punjab market showed 1.67-4.26 % total alkaloid. The yield of Rasaut from B. lycium was found to be 15.4 % w/v and contained about 9.4 % w/v berberine (Anonymous, 1948[9]).

Berberine exists in three tautomeric forms (I-III) in solution. Later on, these tautomeric structures and the evidence for the existence and structures of the ammonium (I) and pseudo-base form (II) were established (Anonymous, 1967[8]). Chemical diversity of various Berberis species is illustrated in Table 3(Tab. 3) (References in Table 3: ***B. aristata:*** Blasko et al., 1982[15]; Potdar et al., 2012[98]. ***B. asiatica***: Chatterjee, 1952[21]; Chandra and Purohit, 1980[20]. ***B. chitria:*** Bhakuni et al., 1968[12]; Hussain and Shoeb, 1958[53]; Ghosh et al., 1993[42]; Yasupov et al., 1990[146]; Ali and Khan, 1978[6]. ***B. lycium:*** Ikram et al., 1996[57]; Miana, 1973[86]; Datta et al., 1976[29]; Leet et al., 1983[75]; 1982[74]. ***B. pachycantha: ***Tomita and Yong, 1960[135]; Du and Francis, 1974[33]. ***B. concinna:*** Tiwari and Masood, 1977[129]; 1978[130]. ***B. corearia:*** Tiwari and Masood, 1979[131]; Majumdar and Saha, 1978[81]; Vereskovskii and Sapiro, 1985[138]. ***B. vulgaris:*** Wierzchowski and Budicz, 1969[143]; Parlamarchulk et al., 1973[96]; Suau et al., 1998[126]. ***B. kawakamii:*** Tsang-Hsiumg and Lu, 1960[136]. ***B. mingetsensis:*** Tsang-Hsiumg and Lu, 1960[136]. ***B. calliobotrys: ***Hussain and Shamma, 1980[55]. ***B. orthobotrys:*** Hussain and Shamma, 1980[55]. ***B. umbellata:*** Masood and Tiwari, 1981[83]. ***B. brandisiana:*** Hussain et al., 1986[54]. ***B. pseudoumbellata:*** Pant et al., 1986[95]. ***B. floribunda: ***Chatterjee et al., 1953[22]. ***B. laurina:*** Falco et al., 1969[36]; 1968[35]; Krets, 1956[69]. ***B. baluchistanica: ***Shamma et al., 1974[109]; 1972[110]. ***B. amursensis: ***Tomita and Kugo, 1955[133]. ***B. thunbergii: ***Tomita and Kikuchi, 1956[132]. ***B. tschonoskyana:*** Tomita and Kugo, 1956[134]. ***B. koreana: ***Pavel, 1965[97]. ***B. tabiensis:*** Quevedo et al., 2008[101]. ***B. coletioides:*** Fajardo et al., 2009[34]. ***B. waziristanica: ***Hussain, 1992[52]).

## Conclusion and future prospective

During the last few decades there has been an increase in the study of medicinal plants and their traditional use in different parts of the world. Reports of the folk medicine followed by critical scientific evaluation have given to the world newer sources as corrective, preventive and upto some extent curative measures in various diseases. *Berberis* species are among the most important traditional herbs with a vast array of pharmacological activities. The present review summarizes the taxonomic, ethno-botanical, pharmacognostic, photochemical and pharmacological claims of *Berberis* species. Literature on Phyto-chemistry reveals that the species are rich in alkaloids, of which biologically active 'Berberine' is the major and potential one. 

This review is a comprehensive documentation of various species belonging to this genus and their therapeutic potentials in the present context. Previous pharmacological studies on *Berberis* and its isolated alkaloids revealed more potential towards cardio vascular, hepato-protective, antimicrobial and anticancer activities. Recent trend in research on *Berberis* species, however directed the workers to focus more towards oncology, toxicological studies and clinical trials. This review will be useful for researchers to approach the newer avenues by exploring varied pharmacological activities like anti diarrheal, antispasmodic, anti malarial, etc., which in turn will be more beneficial in developing myriads of scientifically validated herbal formulations containing naturally occurring biodynamic compounds.

## Acknowledgements

Authors are thankful to the Director, CSIR-NBRI for providing all the facilities. They are also thankful to Dr. KN Nair for his contribution in language check in this review.

## Figures and Tables

**Table 1:**
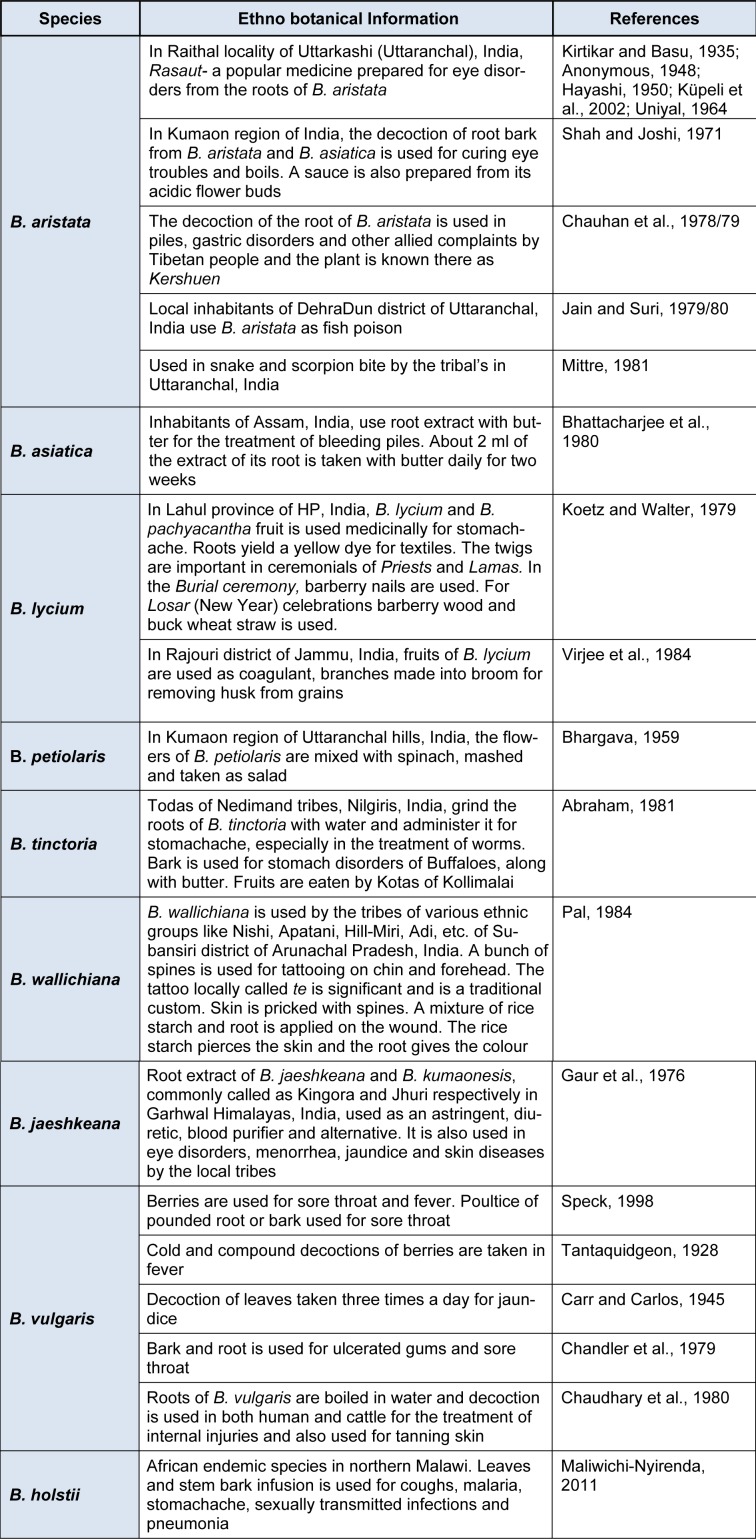
Ethno botanical uses of different species of *Berberis*

**Table 2:**
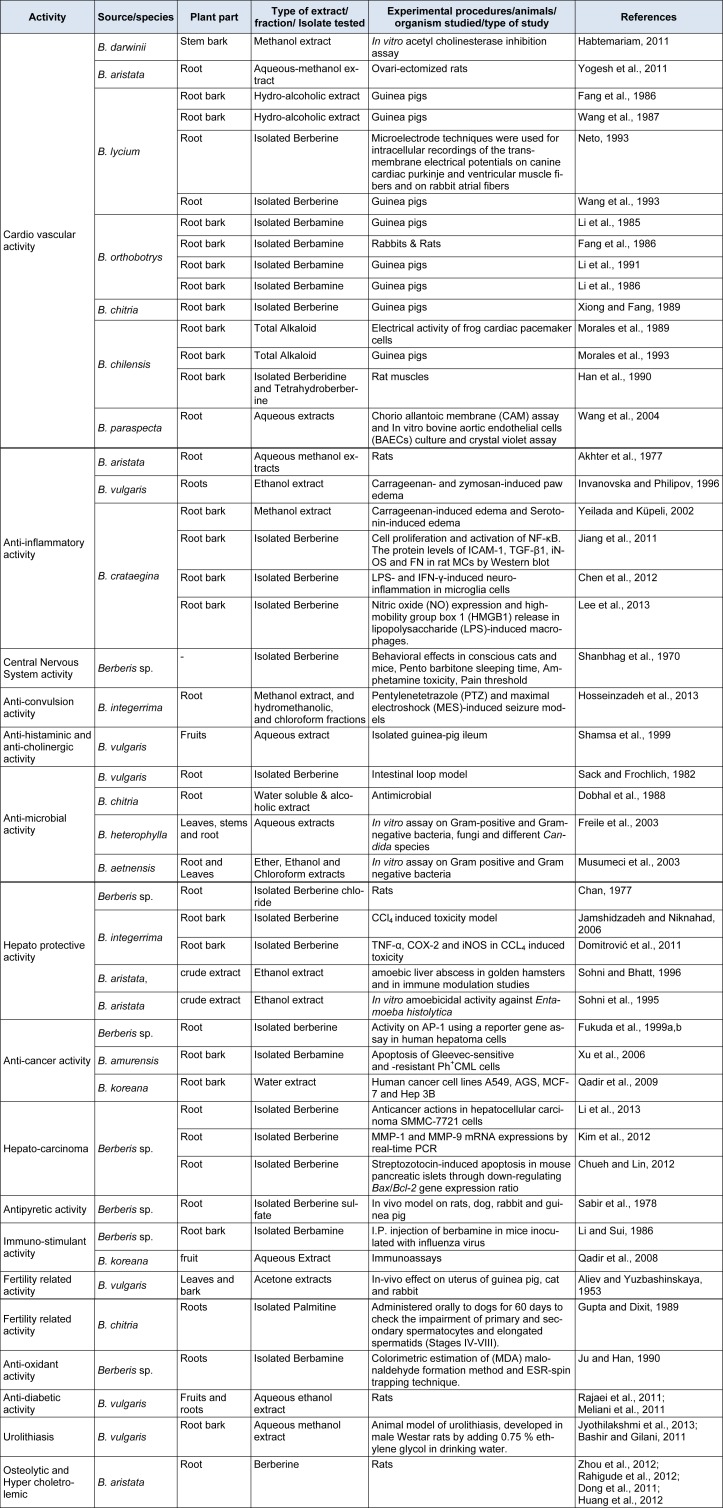
Pharmacological activities of various *Berberis *species

**Table 3:**
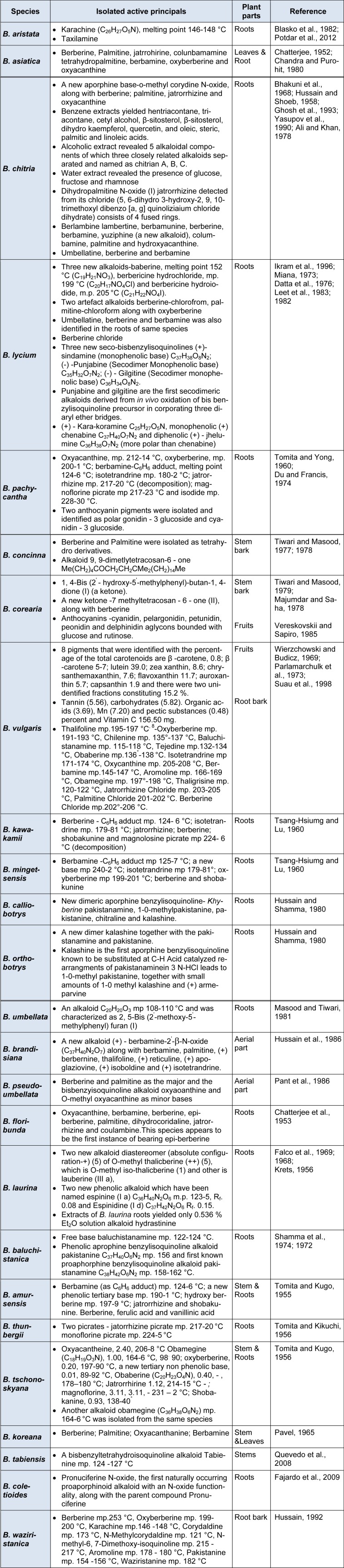
Phyto constituents of various *Berberis* species
